# Exploring Health Care Professionals’ Perspectives on Using Video Feedback and Movement Analysis to Facilitate Physical Functioning for Older Adults Living at Home: Co-Design Approach

**DOI:** 10.2196/73527

**Published:** 2025-11-06

**Authors:** Jessica Olovsson, Mirjam Ekstedt, Cecilia Fagerström, Patrick Bergman, Sofia Backåberg

**Affiliations:** 1 Department of Health and Caring Sciences Faculty of Health and Life Sciences Linnaeus University Växjö/Kalmar Sweden; 2 Department of Learning, Informatics, Management and Ethics Karolinska Institutet Stockholm Sweden; 3 Department of Research Region Kalmar County Kalmar Sweden; 4 Department of Medicine and Optometry Faculty of Health and Life Sciences Linnaeus University Kalmar Sweden

**Keywords:** physical functioning, eHealth, older adults, motivation, video feedback, rehabilitation

## Abstract

**Background:**

Maintaining and motivating physical functioning among older adults has substantial health-related benefits, such as reducing the risk of falls and increasing the opportunities for independent living. Supporting preventive actions among older adults also has socioeconomic relevance. Previous studies have shown that digital tools involving video feedback can facilitate reflection and learning by encouraging active engagement.

**Objective:**

This study aimed to explore health care professionals’ experiences of using a video-based tool as part of the rehabilitation to facilitate physical functioning among older adults (aged ≥65 years) living at home.

**Methods:**

An experience-based co-design approach was used, involving 20 health care professionals. Nine iterative workshops were conducted, followed by 9 group interviews held between 2022 and 2023. The data were analyzed using reflexive thematic analysis.

**Results:**

The results from this study captured the experiences of health care professionals using a video-based tool to facilitate physical functioning in older adults living at home. The participants described focusing on supporting patient commitment, creating a shared language to enhance collaboration in the rehabilitation process, and navigating barriers to adopting the video-based tool in practice.

**Conclusions:**

From the perspective of health care professionals, video feedback has the potential to improve movement performance in daily activities and may play a crucial role in providing motivation and promoting sustainable physical functioning among older adults. Clinical recommendations include training health care professionals to introduce video feedback in a patient-centered manner and using it to foster shared communication that promotes professional development and patient engagement. Further research is needed to assess the impact of video feedback on older adults’ health outcomes and to identify strategies for implementation in complex rehabilitation needs.

## Introduction

As the proportion of older adults in the population increases, there is a growing need for interventions that effectively support their physical functioning [1]. Physical functioning is significantly associated with an individual’s capacity to engage in activities of daily living. This becomes particularly important in older age, when maintaining adequate levels of physical functioning is vital for ensuring independence [2,3]. Remaining physically active despite advancing age is crucial for sustaining physical and mental health and has a preventive effect on major health conditions such as coronary artery diseases [4,5]. Regular physical activity can help prevent falls, which may otherwise lead to severe physical injuries such as fractures. This in turn may lead to fear of falling or social isolation. Early support from health care professionals, for example, rehabilitation staff, to regain physical function is therefore particularly important [6,7]. According to the World Health Organization, adults aged 65 years and above should complement their daily activities with individualized interventions that enhance balance, mobility, and strength [5,8]. Today, a large proportion of older adults reside at home. Older adults who receive home care report various barriers to physical activity, such as illness, a perception of being too old, and a fear of falling [9,10]. As people age, levels and patterns of physical activity often shift. Few people aged 65 years and above achieve the recommended levels of physical activity. According to Swedish national data, older adults spend approximately 10 hours per day sitting [11]. Relatives and close friends are essential in supporting older adults to maintain independence and physical function. In parallel, health care professionals in multidisciplinary teams play a crucial role in promoting these outcomes by providing specialized expertise and support [12]. Integrating physical activity into everyday life so that it becomes part of the daily routine is crucial for maintaining motivation [13,14].

Previous studies support the use of eHealth interventions to increase physical functioning [15,16]. Although eHealth interventions have been found to have positive outcomes and effects on physical activity among older adults, a gap in knowledge remains regarding their link to long-term health and behavioral changes [16,17]. Success factors for eHealth interventions that promote physical activity include support from health care professionals, self-monitoring, and social networking. Obstacles to using eHealth in health care include limited resources, the absence of organizational strategies, and a lack of knowledge [17,18]. This underlines the importance of acknowledging health care professionals’ perspectives when developing and implementing eHealth interventions. Previous research shows that the motivation to learn new skills and remain active may be supported by interactive communication with health professionals [19]. Although both health care professionals and patients value eHealth in rehabilitation, their differing priorities (clinical functionality for professionals and social connectedness for patients) underscore the importance of designing solutions that address both emotional and functional motivation [20,21]. In a study by Kraaijkamp et al [22], health care professionals were found to perceive eHealth as being more complex for patients than for themselves. Therefore, tailored implementation strategies for eHealth interventions are required, with health care professionals’ perspectives being essential to identifying and overcoming barriers. Even if eHealth interventions prove to be useful, implementation of work processes in health care that meet the patients’ needs for support is challenging [23].

Research conducted in contexts outside of rehabilitation for older adults has demonstrated that video feedback can facilitate self-reflection by promoting active engagement and drawing attention to various aspects of movement. When combined with self-controlled feedback, where learners actively participate in the feedback process, video feedback tools have shown promising outcomes among younger participants [24-26]. Additionally, video feedback can enhance understanding of self-care management and serve as a valuable complement to traditional health care services [21]. However, despite these promising findings, the integration of interactive video feedback into rehabilitation at home remains rare. Furthermore, there is a notable gap in research examining the use of video feedback within rehabilitation settings for older adults, particularly from the perspective of health care professionals. Their insights are crucial for understanding practical implementation, perceived value, and potential barriers in clinical practice. This study aimed to explore health care professionals’ experiences of using a video-based tool as part of rehabilitation to facilitate physical functioning among older adults living at home.

## Methods

### Study Design

**Figure 1:**
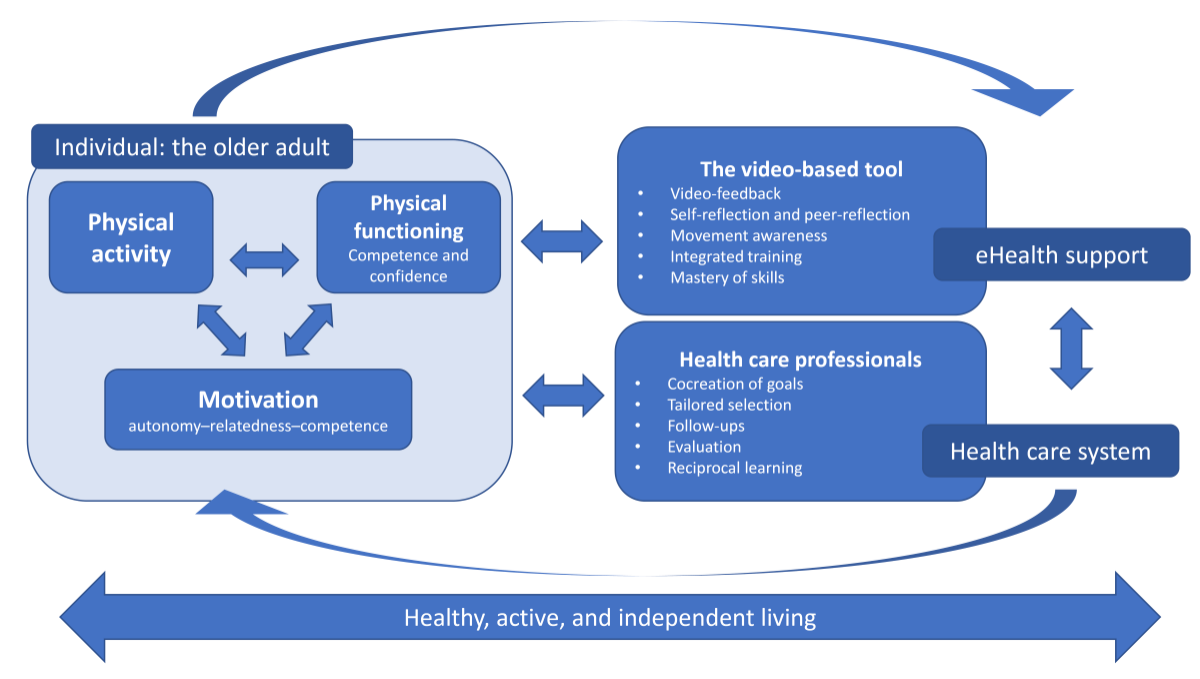
The preliminary program theory.

The methodological approach was guided by the Medical Research Council’s framework for developing and evaluating complex interventions [27]. The study adopted a participatory research design to engage stakeholders in developing the intervention. An experience-based co-design approach was applied [28], involving iterative workshops and group interviews with elements of think-aloud techniques [29]. The video feedback intervention was initially developed based on theoretical foundations and previous research. Following the Medical Research Council’s framework, the primary focus of this study was to refine the intervention and retest the tentative program theory (Figure 1), identify key uncertainties, and adapt it to a new context (ie, rehabilitation at home for older adults). To address these steps, key stakeholders were engaged as co-design collaborators, including health care professionals, for example, physiotherapists, occupational therapists, and rehabilitation assistants involved in rehabilitation for older adults aged ≥65 years. The study was conducted as part of the larger research project *Confident in every step*, which was guided by a tentative program theory based on self-determination theory (SDT) [30]. The preliminary program theory includes (1) the patient and their resources (eg, knowledge, skills, and social connections); (2) the health care provider and their resources; (3) a digital tool enabling access to supportive information, learning, and feedback; and (4) the interactions, intentions, and capabilities of all parties to achieve health outcomes valued by older adults and supported by health care providers. These interactions are illustrated in Figure 1.

### The Video-Based Tool

The video-based tool MOVEImprove (Savvy Knowledge Corporation) was used to support video feedback, structured analysis, and self-reflection on movement performance through guiding questions for each movement component. The platform was developed by researchers at the University of Calgary, and contents were developed by researchers at Linnaeus University. The video-based tool is compatible with mobile phones and tablets and allows users to record each other with the aim of improving physical skills. Assessments and reflections take place in pairs. In this study, health care professionals recorded patients performing exercises from 4 themes targeted at older adults: *balance for older adults*, *daily life movements*, *breathing exercises*, and *picking up from the floor*. The latter 2 themes were co-designed by researchers at Linnaeus University in earlier phases of the larger research project [21,31].

### Data Collection

A total of 20 health care professionals from 3 municipal and regional primary care organizations in Sweden were recruited through convenience sampling. Participants involved in rehabilitation for older adults (Table 1) were invited face-to-face by representatives within their respective organizations.

In total, 9 workshops were conducted with 6 to 10 participants in each session; 2 were held digitally, whereas the rest took place at the participants’ workplaces. Each organization participated in recurring workshops, followed by group interviews. Group interviews were semistructured and based on workshop outcomes. Co-design workshops and group interviews were conducted between 2022 and 2023, with 2 to 5 sessions per organization (Table 2).

**Table 1:** Demographic characteristics of participants (N=20).

Characteristic		Participants
Age (years), range		23-61
**Gender, n (%)**		
	Women	18 (90)
	Men	2 (10)
Professional experience (years), range		1-32
**Professions, n (%)**		
	District nurse	2 (10)
	Physiotherapist	9 (45)
	Occupational therapist	4 (20)
	Rehabilitation assistant	2 (10)
	Medical officer for rehabilitation	2 (10)
	Home care manager	1 (5)

**Table 2:** Data collection: workshops and subsequent group interviews.

Workshop (organization and format)	Participants by profession, n
Workshop 1 (organization A, in person)	1 MAR^a^, 1 RA^b^, 1 OT^c^, and 2 PTs^d^
Workshop 2 (organization A, online)	1 MAR, 1 RA, 1 OT, and 2 PTs
Workshop 1 (organization B, in person)	1 OT and 4 PTs
Workshop 2 (organization B, online)	3 PTs
Workshop 1 (organization C, in person)	1 MAR, 2 OTs, 1 PT, and 1 HM^e^
Workshop 2 (organization C, in person)	1 OT, 2 PTs, 1 RA, 1 DS^f^, and 1 HM
Workshop 3 (organization C, in person)	1 MAR, 2 OTs, 3 PTs, 1 RA, and 2 DSs
Workshop 4 (organization C, in person)	1 MAR, 2 OTs, and 2 PTs
Workshop 5 (organization C, in person)	1 MAR, 2 OTs, and 3 PTs

^a^MAR: medical officer for rehabilitation.

^b^RA: rehabilitation assistant.

^c^OT: occupational therapist.

^d^PT: physiotherapist.

^e^HM: home care manager.

^f^DS: district nurse.

The first author (JO), together with the last author (SB), facilitated all the workshops and group interviews. Each session, including interviews, lasted 60 to 180 minutes. All sessions were digitally recorded, and reflective notes taken by the research team supported and validated the data during analysis. During the iterative workshops, participants tested the video-based tool with each other and reflected on the usability of the tool, including reflections considering using it in a new context, that is, rehabilitation for older adults. Between sessions, health care professionals were provided with tablets to use the video-based tool with patients. Patient selection was based on the following inclusion criteria: aged 65 years or more, living at home, and receiving primary care or a newly implemented home care service.

Decisions were made in line with the Medical Research Council’s framework by the research team and engaged stakeholders to review the progress and determine the next steps for the workshops and interviews. The co-design process resulted in a refined intervention, that is, a structured user guide for the video-based tool, including refined inclusion criteria of the patient population and a summary of identified areas of use. Through co-design with health care professionals, additional functionalities were introduced, encompassing comparative video analysis and slow-motion playback to improve the precision and clarity of movement execution when using the video-based tool.

### Data Analysis

Workshop and interview recordings were transcribed and verified by the first author (JO). Data were analyzed using the reflexive thematic analysis by Braun and Clarke [32,33], following an inductive approach. Transcripts were organized in NVivo (QSR International) to structure the data and support data management and traceability, whereas the thematic analysis was conducted manually in a step-by-step manner. Reflexive thematic analysis involved 6 steps. In step 1, the first author familiarized herself with the data through repeated readings. In step 2, initial codes were generated by the first author using open coding. In step 3, revised codes were collected. Theme development was discussed with coauthors (SB, ME, and CF), and subthemes were collaboratively refined into initial themes by the full research team (JO, SB, ME, CF, and PB). In step 4, overarching themes were reviewed and further discussed. The process involved iterative movement between quotations, coded segments, and the full dataset to ensure coherence and contextual grounding. Themes and subthemes were subsequently reviewed and refined to facilitate reflexive engagement with the data and to ensure that quotations had coherence with the original data. Step 5 involved defining and naming themes. Examples of the analysis process are presented in Table 3. Step 6 involved writing a coherent text that presents themes, including interpretation of the data [32,33].

**Table 3:** Examples of the reflective thematic analysis process.

Data extract	Code	Subtheme	Theme
“Well, it’s been an eye-opener, because I might have felt that no, I don’t want to use an iPad and such, because I felt that I was maybe a bit afraid that digitalization would distance me from the patient...or like my feeling from the start when we’ve talked about needing to digitalize more and so on, that it would mean I’m here with my screen and the patient is at home with their screen.” [Participant 13]	Concern among health care providers that digital tools create distance	Navigating thresholds to adopting the tool	A new way of working
“I think where we filmed was more to build her confidence, or whatever you want to call it, to see that she actually managed it. There weren’t many days in between, but there was still a clear improvement, and she got to see ‘Well, I can actually do it.’” [Participant 19]	Film to build self-confidence	Clarifying sustainable movements and changes over time	Supporting patient commitment

### Ethical Considerations

This study adhered to the principles of the Declaration of Helsinki [34] and was approved by the Swedish Ethical Review Authority (2023-00276-01). Participation was voluntary, and participants were informed that they could withdraw at any time without providing a reason. All participants received both verbal and written information about the study, including assurances that participation would not affect their current work situation or rehabilitation process. Written and verbal informed consent was obtained from all participants prior to data collection. Participants were made aware of the study’s purpose, procedures, and their rights. Videos recorded in the clinic that included patients were deleted immediately by the responsible health care professionals to ensure privacy. All data were handled confidentially, and no identifiable information was retained or published. Participants did not receive any financial or material compensation for their participation. Data collection was conducted with attention to diversity in gender and cultural representation to ensure inclusivity and relevance.

## Results

The analysis resulted in 3 overarching themes that described health care professionals’ experiences of using a video-based tool to facilitate physical functioning in older adults within municipal and regional primary health care settings (Textbox 1 [32,33]).

**Overview of the 3 themes and 6 subthemes identified through the reflexive thematic analysis, as described by Braun and Clarke.**

**Supporting patient commitment**

- Visualizing movement patterns and movement quality
- Clarifying sustainable movements and changes over time
- Enabling reflection and body awareness

**Creating a shared language**

- Facilitating collaboration with the patient
- Cocreation in the rehabilitation process

**Navigating thresholds to adopting the tool**

- Identifying the right patient
- Unfamiliarity: a new way of working

### Supporting Patient Commitment

The video-based tool was perceived as a clinical pedagogical aid that enhanced patient commitment. Health care professionals described the video-based tool as a valuable method for illustrating movement quality to patients in a clear and accessible manner. Visual feedback enabled patients to observe their own movement patterns, fostering reflection and self-assessment. However, responses varied: some patients were overly generous in their evaluations, while others were highly self-critical, highlighting the subjective nature of movement perception. The video-based tool was perceived as a significant advancement over traditional methods such as mirror-based observation, as the video feedback allowed for retrospective analysis and more objective evaluation of movement patterns. This facilitated detailed discussions between professionals and patients. It also allowed for objective measurements and the ability to review movements retrospectively. Therefore, it could serve as a potential evaluation resource, particularly useful for “before-and-after” comparisons in areas such as mobility, balance, and movement quality. Health care professionals described the video-based tool as a valuable instrument for documenting progress throughout the rehabilitation process. By enabling movement performance over time, it allowed patients to see how close they were to achieving their rehabilitation goals. This functionality supported professionals in tracking changes and making informed decisions about ongoing care. A participant reflected as follows:

“Well, I’m in a lot of pain, it doesn’t work, I still can’t walk.” No, but a week ago you could only sit up for 15 seconds and now you’re sitting here without holding on to anything. So, it can be an opportunity for them to see that for themselves.

Participant 1

By providing visual evidence of functional improvement, the tool contributed to strengthening patients’ confidence in their physical capabilities:

I think where we filmed was more to build her confidence, or whatever you want to call it, to see that she actually managed it. There weren’t many days in between, but there was still a clear improvement, and she got to see “Well, I can actually do it.”

Participant 19

Health care professionals reported that the use of the video-based tool contributed to patients feeling acknowledged within the rehabilitation process. This sense of recognition was linked to the commitment demonstrated by professionals who actively engaged with the tool to support individualized care. The visual feedback provided by the tool was perceived as a means of affirming each patient’s efforts and progress, thereby strengthening the therapeutic relationship. Some rehabilitation professionals were surprised by the potential of video technology in clinical practice, particularly in its ability to enhance patient engagement and interprofessional collaboration. However, they also emphasized the importance of identifying key facilitators to ensure successful implementation. These included effective time management, collaborative work in pairs, and strategic planning within the clinical team to address organizational challenges and sustainably integrate the tool into everyday practice. Health care professionals described the video-based tool as a valuable means of fostering patient reflection and enhancing body awareness. By enabling repeated observation and discussion of movement performance, the tool supported a deeper understanding of functional abilities and limitations. The visual feedback facilitated detailed reflection, allowing patients to recognize subtle aspects of their movements that might otherwise go unnoticed. Health care professionals noted that viewing recorded movements could evoke memories of previous physical capabilities, particularly in individuals recovering from injury. This process was believed to be instrumental in reconnecting patients with familiar movement patterns, thereby supporting the restoration of motor function. The tool also provided opportunities to revisit and reinforce therapeutic practices from earlier stages of life or previous rehabilitation experiences. A participant described recollecting an encounter with a patient, who stated,

“Well, this is exactly how I’ve done it before.” And then she started. “This is how I used to exercise,” and it felt like she found her muscle memory again.

Participant 11

Through this reflective process, the video-based tool contributed to active reflection and engagement in rehabilitation. Although the video-based tool was generally perceived as a valuable aid in enhancing body awareness and movement reflection, health care professionals also noted that its use could inadvertently lead to altered movement performance. The act of being filmed heightened patients’ self-consciousness, prompting some to modify their movements in ways that were not representative of their habitual motor patterns. This performative response—driven by a desire to appear more competent or polished on video—was seen as a potential limitation, as it could decrease the authenticity of the movement performance.

### Creating a Shared Language

Health care professionals found the video-based tool helpful in supporting learning and documenting patient information, particularly when multiple people were involved. This was evident in various contexts, including the patient-rehabilitation professional relationship. Health care professionals emphasized the importance of mutual understanding between professionals and patients in the rehabilitation process. The video-based tool was perceived as a valuable aid in fostering this collaboration, enabling clinicians to visually demonstrate specific movements, exercises, and progress. By showing tangible improvements, professionals could enhance patient collaboration. A participant shared the following example from clinical practice:

Well, look, this is how it was three weeks ago; now you [the patient] can actually do this, and you [the patient] can do it yourself. Now the caring staff only needs to stand by.

Participant 12

This visual feedback supported shared decision-making and strengthened the therapeutic alliance, allowing patients to take a more active role in their rehabilitation. The video-based tool was described as a facilitator of cocreation within the rehabilitation process, supporting professional collaboration. Health care professionals emphasized its value for internal learning and consensus building among rehabilitation staff, helping teams establish a shared understanding of patient progress and therapeutic strategies. The tool also served as a resource for external caregivers, such as home care staff, by visually demonstrating appropriate levels of assistance. This was seen as particularly important in preventing overassistance, which could inadvertently limit patient autonomy and rehabilitation outcomes. Observing how exercises were performed across different settings, whether in health care facilities or at home, was considered especially beneficial during handovers. However, health care professionals noted that incompatible journal systems across organizations posed a barrier to seamless information sharing. The ability to store a digital history of movement performance was viewed as a key advantage, enabling professionals to track progress and support individualized care planning. A participant explained the following:

It’s quite easy for a colleague to take over when there’s a digital history, as they can look at the first and last videos and see what they’ve been working on, and what we should continue with if needed.

Participant 1

### Navigating Thresholds to Adopting the Tool

Rehabilitation professionals encountered various challenges when integrating the video-based tool into clinical practice and described this as a process of overcoming obstacles. Establishing a therapeutic relationship was described as a key facilitator for the effective use of the video-based tool. As such, its implementation was typically deferred until after the initial patient contact, allowing time to build trust and assess suitability. Health care professionals identified that the tool was most beneficial for patients who could both articulate and reflect on their movement difficulties—an ability considered uncommon in older adult care settings. Cognitive capacity was also noted as an important factor, though views on this varied. Although some professionals regarded cognitive impairment as a barrier, others found that it did not necessarily preclude meaningful engagement with the tool. The video-based tool was particularly valued in cases involving frail older adults with limited energy, where repeated movement demonstrations were challenging. In such contexts, the ability to record and review performance across sessions enabled more detailed analysis and individualized care planning. The integration of video-based tools into rehabilitation practice was described as a shift that was novel and, at times, challenging. Health care professionals expressed concerns regarding data security and the ethical management of visual recordings within medical documentation. In addition, some professionals feared that digitalization might compromise the therapeutic relationship by creating an emotional or physical distance between clinician and patient. A participant reflected as follows:

Well, it’s been an eye-opener, because I might have felt that no, I don’t want to use an iPad and such, because I felt that I was maybe a bit afraid that digitalization would distance me from the patient...or like my feeling from the start when we’ve talked about needing to digitalize more and so on, that it would mean I’m here with my screen and the patient is at home with their screen.

Participant 13

Initial use of the tool also prompted safety-related adaptations. Health care professionals reported a need to remain physically close to patients or work in pairs, particularly during early sessions, to mitigate risks such as falls and to deal with the unfamiliarity of the method. Preconceived notions about older adults, such as discomfort with being filmed or limited digital literacy, were common but often challenged through practice. These findings highlight the importance of balancing innovation with relational and safety considerations in clinical implementation.

## Discussion

### Principal Findings

This study explored health care professionals’ experiences with a video-based tool designed to facilitate physical functioning in older adults living at home. Participants highlighted its role in fostering patient engagement, facilitating a shared language in collaborative rehabilitation. The video-based tool was perceived as a motivational source that could enhance patients’ confidence in movement. It was also perceived to improve interprofessional communication, particularly during handovers and collaborative planning, by demonstrating physical functioning and rehabilitation needs. Reflecting on video recordings enabled shared understanding of rehabilitation goals. The study’s findings align well with SDT, as described by Ryan and Deci [30], which emphasizes the importance of 3 basic psychological needs in fostering intrinsic motivation and well-being: autonomy, competence, and relatedness. *Autonom*y is supported when individuals feel they are the origin of their actions. The video-based tool empowered older adults to engage with their rehabilitation in a way that felt meaningful. It also allowed older adults to observe and reflect on their own movements, giving them a sense of control and ownership over their rehabilitation. Seeing their own progress visually could help older adults set personal goals in the rehabilitation process. *Competence* is fostered when individuals feel capable of achieving desired outcomes. Participants noted that the video-based tool allowed patients to observe incremental improvements, providing clear evidence of progress. Capturing positive aspects of performance helped reinforce a sense of achievement, even in small steps. Conversely, when the participants did not achieve the desired outcomes, video feedback failed to enhance their sense of competence and may have adversely affected their perceived capability. Therefore, visual feedback could be especially powerful for older adults who struggle with self-efficacy due to age-related decline. *Relatedness* is fulfilled when individuals feel connected to others. Shared use of the video-based tool fostered a sense of partnership in the rehabilitation process, highlighting the importance of involving health care professionals in using video feedback with older adults. Reflecting on video recordings together could foster mutual understanding. The video-based tool also facilitated interprofessional communication, which supports collaborative rehabilitation. The findings from this study support the idea that use of the video-based tool in close collaboration with health care professionals can enhance intrinsic motivation in older adults by satisfying the core psychological needs outlined in the SDT. The findings also support the larger research project’s preliminary program theory (Figure 1), suggesting that video feedback and movement analysis can promote sustained physical functioning and physical activity, which in turn promotes active and healthy aging. Furthermore, the results provide a deeper understanding of the preliminary program theory, offering potential for its refinement and expansion, particularly in relation to how video feedback can support psychological aspects of rehabilitation, such as confidence and motivation.

### Comparison With Previous Work

Video-based feedback has proven especially valuable for older adults by highlighting incremental improvements and reinforcing positive aspects of movement. Previous research indicates that aging is often accompanied by psychological stress, making visual confirmation of physical performance especially impactful and motivating [4,35,36]. The findings of this study highlight the importance of motivation in rehabilitation, particularly when progress is made visible through structured feedback [21,37]. Health care professionals emphasized the need to identify appropriate target groups for the tool. As more patients were included and positive outcomes were observed, the perceived applicability of the tool broadened. Although cognitive ability was considered relevant, cognitive impairment was not viewed as a barrier, which was seen as an encouraging stance. This contrasts with the frequent exclusion of cognitively impaired older adults from research, limiting the development of inclusive, evidence-based interventions [38]. By involving this population, the study contributes to more equitable rehabilitation research. eHealth interventions offer potential for promoting healthy aging but require thoughtful implementation [31,39]. The video-based tool showed promise for early detection of functional decline through structured movement analysis; however, the mechanisms underlying this effect and its comparative effectiveness remain unclear. Preventive interventions for community-dwelling older adults have yielded positive health outcomes [40]. The video-based tool that was refined in our study may contribute meaningfully to preventive strategies and support such efforts. However, challenges remain in implementing digital tools in rehabilitation, including concerns about data security, reimbursement, legal frameworks, and digital literacy among older adults [41]. Previous studies show that although physiotherapists value evidence-based digital tools for improving access and self-management, they also express concerns about reduced interaction and patient adherence. Successful implementation requires clear guidelines, training, and a user-friendly design [42]. While our study acknowledged similar findings, further investigation is warranted to explore the diversity and potential contradictions within these perspectives in the context of rehabilitation for older adults.

### Strengths and Limitations

Credibility was enhanced through the inclusion of diverse samples from multiple settings, as elaborated in the criteria for qualitative research by Lincoln and Guba [43]. Workshops and interviews were conducted by professionals with different clinical backgrounds (physiotherapy and nursing). This diversity was expected to provide a range of perspectives. All interviews were transcribed verbatim and verified by the first author, with quotations used to support findings. Codes, subthemes, and themes were reviewed iteratively to enhance confirmability, including discussions of conceptual depth. Dependability was supported by consistent use of a semistructured interview guide and a single transcriber. The authors maintained reflective journals to document insights and ensure analytic consistency over time.

Transferability was addressed by clearly describing participants, data collection methods, and timing, allowing findings to be applied in similar rehabilitation contexts. Open-ended questions, think-aloud techniques, and repeated workshops promoted participant engagement. However, group interviews may have limited dissenting views due to hierarchical dynamics, although no participants were in dependent relationships. Interviews with multiprofessional groups may have influenced responses, which should be considered when interpreting the results. The study encompassed 3 workplaces, with varying numbers of workshops and intervals, which may have affected the depth of engagement with the video-based tool. Although most sessions were conducted in person, 2 were held digitally at participants’ request, potentially limiting interactions with the research team.

### Conclusions

From the perspective of health care professionals, experiences of using video feedback for older adults to support patient commitment, foster a shared language, and navigate thresholds for adopting the video-based tool into practice have shown potential to motivate enhanced movement performance and could therefore play an important role in sustainably facilitating physical functioning. However, its implementation requires considerations of ethical data management and potential influence on the therapeutic relationship. For patients, it may offer a way to engage more actively in their rehabilitation. Responses to being filmed ranged from improved confidence to altered movements, revealing the subjective nature of movement perception. Therefore, clinical recommendations include training health care professionals to introduce video feedback in a manner that is patient centered, emphasizing its role in supporting motivation rather than serving solely as an evaluative tool. Furthermore, video feedback should be used to establish a shared communicative framework that enhances professional development and facilitates constructive dialogue with patients regarding their rehabilitation progress. These insights offer a meaningful contribution to the preliminary tentative program theory, indicating that the mechanisms proposed, such as self-observation, reflection, and motivation, may be effective in rehabilitation for older adults. However, further research is warranted to investigate the effect and impact of video feedback on older adults’ physical abilities and related health outcomes. Future studies should be conducted particularly in settings where older adults often face significant challenges in maintaining physical function and independence, such as during care transitions or when presenting with complex rehabilitation needs.

## Abbreviations

SDT: self-determination theory

## Data Availability

The data generated or analyzed during this study are not publicly available as participants did not consent to public availability. Anonymized data are available from the corresponding author on reasonable request (aggregated data in Swedish).
